# Reticulocyte Hemoglobin Equivalent: Diagnostic Performance in Assessment of Iron Deficiency in Patients with Hypothyroidism

**DOI:** 10.1155/2021/9071057

**Published:** 2021-11-12

**Authors:** Wardah Aslam, Maryam Habib, Saeeda Aziz, Madiha Habib

**Affiliations:** ^1^Nuclear Medicine Oncology and Radiotherapy Institute, Islamabad, Pakistan; ^2^Shifa College of Medicine (STMU), Islamabad, Pakistan; ^3^University of Malaya, Kuala Lumpur, Malaysia

## Abstract

**Introduction:**

Iron deficiency affects approximately 30% of the world population and is frequently encountered in hypothyroid patients. Early recognition and prompt treatment of iron deficiency in hypothyroid patients lead to a favorable outcome. The aim of this study is to prove the usefulness of reticulocyte hemoglobin equivalent (Ret-He) as a reliable and effective tool in diagnosis of iron deficiency in hypothyroid patients.

**Materials and Methods:**

154 patients with hypothyroidism were included in the study. They were divided into 4 groups. Group 1 included 66 hypothyroid patients without iron deficiency. They were taken as controls. Group 2 included 66 hypothyroid patients with iron deficiency anemia (IDA). Group 3 included 12 hypothyroid patients with iron deficiency but without anemia (ID). Group 4 included 10 hypothyroid patients which had concomitant iron deficiency with anemia of chronic disorder (ACDC). Ret-He was measured by analyzing blood samples on System XN 350. Thyroid profile, serum ferritin, and biochemical data were measured by an automated analyzer. Statistical analysis was performed by using SPSS 23.

**Results:**

Ret-He was significantly lower with (*p* < 0.001) in group 2 (hypothyroid patients with IDA), group 3 (hypothyroid patients with ID), and in group 4 (hypothyroid patients with ACDC) as compared to controls in group 1 (hypothyroid patients without iron deficiency). After ROC analysis area under the curve (AUC) of Ret-He for hypothyroid patients with IDA was 0.96 at cutoff 28.5 pg with sensitivity of 93% and specificity of 90%. AUC of Ret-He in the hypothyroid group with ACDC was 0.99 at cutoff 30.8 pg with sensitivity of 90% and specificity of 90%. AUC of Ret-He in hypothyroid patients with ID was 0.97 at cutoff 31.7 pg with sensitivity of 91% and specificity of 70%.

**Conclusion:**

Ret-He is a reliable, rapid, and cost-effective tool for diagnosing iron deficiency in hypothyroid patients.

## 1. Introduction

Anemia is a global health problem affecting about one-third of population worldwide [1]. Iron deficiency anemia is one of the most common causes of anemia. Approximately half of all the cases of anemia throughout the world are due to iron deficiency. It affects all age groups, particularly children and women of childbearing age. It remains as one of the leading causes of morbidity and mortality. Iron deficiency may develop before development of frank anemia, and it may exist in combination with other anemias. Worldwide iron deficiency remains undiagnosed in most of the patients admitted with different illnesses in hospitals, especially before the development of frank iron deficiency anemia [2–5]. Iron deficiency is a state in which body is unable to meet its requirements due to unavailability of iron. It may be in the form of absolute iron deficiency, in which there is insufficient iron in body stores for adequately matching the demand by erythropoiesis in the bone marrow, whereas functional iron deficiency is a state in which the body contains sufficient or more than sufficient iron, but the iron cannot be mobilized from the stores in the reticuloendothelial system to meet the demand for the erythropoiesis [6]. The other important cause of anemia related to iron metabolism is immune activation causing anemia of chronic disorder [7]. Iron deficiency is commonly seen in hypothyroid patients. This can occur due to a variety of reasons. These reasons include decreased iron absorption from gut because of decreased enzymes and acids, decreased levels of erythropoietin found in hypothyroid patients, and in females with menorrhagia due to hypothyroidism. Many hypothyroid patients are not screened for iron deficiency due to financial constraints of ordering multiple tests and cumbersome ways of diagnosing it particularly in the undeveloped countries. Timely recognition and treatment of iron deficiency in hypothyroid patients can lead to better outcomes with greater and improved response to treatment with thyroxine. Iron deficiency can manifest itself as frank iron deficiency anemia, latent iron deficiency, and iron deficiency in combination with anemia of chronic disorder in hypothyroid patients [8–13]. Diagnosis and investigation of iron deficiency requires many parameters which include serum ferritin, serum iron, total iron binding capacity, serum transferrin, and transferrin saturation [14]. Reticulocytes are immature RBCs that are released from the bone marrow. New parameters have been defined for reticulocyte analysis which measure hemoglobin content of reticulocytes and stages of their development. These parameters include immature reticulocyte fraction (IRF), reticulocyte hemoglobin equivalent (Ret-He), and reticulocyte hemoglobin content (CHr). Ret-He and CHr give information about mean content of hemoglobin in developing reticulocytes and are thus affected earlier in diminished hemoglobin production in iron deficiency and iron deficiency anemia as compared to other parameters. Ret-He can be used as an effective tool to detect iron deficiency in different states and anemias. It can be done on the same sample taken for complete blood count in EDTA anticoagulant. Instead of performing many biochemical tests for detecting iron deficient states, it can be used solely for the identification of iron deficiency. Performing many tests leads not only to increased cost for patients, but some of the tests used for detecting iron deficiency have a limited value in presence of infection and malignancy [15]. Ret-He can be used as a single, rapid, and cost-effective test for determining iron status [16–18]. Many studies have been conducted in the past establishing effectiveness of Ret-He in diagnosing iron deficiency in patients suffering from different disorders. To our knowledge, this is one of the first studies aiming to determine the effectiveness of Ret-He in evaluating iron deficiency with and without anemia in hypothyroid patients. This will lead to timely recognition of iron status in hypothyroid patients resulting in better and effective management and improved outcome in these patients.

## 2. Materials and Methods

This prospective study was conducted at Nuclear Medicine Oncology and Radiology Institute, Islamabad, over a period of 2 years from February 2019 till November 2020.The study protocol was approved according to Ethics Review Board of Nuclear Medicine Oncology and Radiotherapy Institute, Islamabad, on 29^th^ January 2019 with ethical reference no. NORI-2(10)/88. One hundred and fifty-four adult patients with hypothyroidism (based on clinical diagnosis and laboratory confirmation) presenting to the outpatient department were randomly enrolled in the study. Hypothyroid patients with hemoglobinopathies and nutritional deficiency anemia other than iron deficiency were excluded from the study. As the study was conducted based on thyroid profile, CBC reports, and relevant biochemical investigations of patients with no direct contact with the patient, informed consent was not required. Waiver for informed consent was provided by the Ethical Committee and Review Board of Nuclear Medicine Oncology and Radiotherapy Institute, Islamabad. Hypothyroid patients were divided into four groups. Group 1 included 66 hypothyroid patients without iron deficiency. They were taken as controls. Group 2 included 66 hypothyroid patients with iron deficiency anemia (IDA). Group 3 included 12 hypothyroid patients with iron deficiency but without anemia (ID). Group 4 included 10 hypothyroid patients who had concomitant iron deficiency with anemia of chronic disorder (ACDC). The inclusion criteria for hypothyroidism, IDA (males and females), ID (males and females), ACDC (males and females), and control (males and females) are mentioned.

  Hypothyroidism: serum TSH >4.0 mIU/L, T3 <3.1 pmol/L, and/or T4 <12 pmol/L.
  IDA (female): Hb <7.45 mmol/L, serum ferritin <13 ug/L, TIBC >46.36 umol/L, serum iron <11 umol/L, and CRP <5 mg/L
  IDA (male): Hb <8.7 mmol/L, serum ferritin <30 ug/L, TIBC >46.36 umol/L, serum iron <14 umol/L, and CRP <5 mg/L
  ID (female): Hb >7.45 mmol/L, serum ferritin <13 ug/L, serum TIBC >46.36 umol/L, serum iron <11 umol/L, and CRP <5 mg/L
  ID (male): Hb >8.07 mmol/L, serum ferritin <30 ug/L, serum TIBC >46.36 umol/L, serum iron <14 umol/L, and CRP <5 mg/L
  ACDC (female): Hb <7.45 mmol/L, serum TIBC <46.36 umol/L, serum iron >11 umol/L, serum ferritin >13 ug/L, and CRP >5 mg/L.
  ACDC (male): Hb <8.07 mmol/L, serum TIBC <46.36 umol/L, serum iron >11 umol/L, serum ferritin >30 ug/L, and CRP >5 mg/L.
  Control (male): Hb> 8.07 mmol/L, serum ferritin >13 ug/L serum TSH >4.2 mIU/L, T3 <3.1 pmol/L, and/or T4 <12 pmol/L, CRP< 5 mg/L.
  Control (female): Hb >7.45 nmol/L, serum ferritin >13 ug/L, serum TSH >4.2 mIU/L, T3 <3.1 pmol/L, T4 <12 pmol/L, and CRP <5 mg/L.

Thyroid profile was performed on COBAS E602 by using electrochemiluminescence technology. Biochemical parameters were measured using ROCHE COBAS 60000 by using immunoassay (electrochemiluminescence method). CBC parameters including Hb, MCV, RDWCV, RDWSD, and RDWSD were measured by using Sysmex XN 350, which used the cyanide-free SLS method for measuring hemoglobin and the impedance method with hydrodynamic focusing for measuring RBC parameters. Reticulocyte hemoglobin equivalent was measured on the same sample by running Sysmex XN 350 on the retic mode by using the fluorescence flow cytometry method. All the data were analyzed by using SPSS Version 23. Descriptive analysis of the data was performed. Age and gender were statistically comparable between 4 groups. The Kolmogorov–Smirnov and Shapiro–Wilk test revealed no significant departure from normality for the variables. ROC curve was used to find area under curve (AUC) for Ret-He in IDA, ID, and ACDC in hypothyroid patients keeping serum ferritin and transferrin saturation levels <20% as gold standard.

## 3. Results

**Figure 1:**
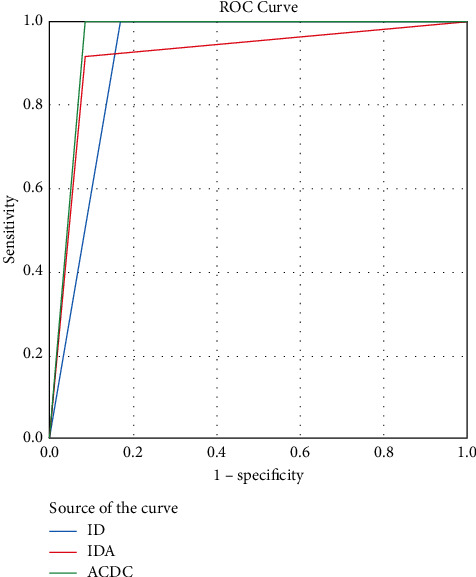
Comparison of ROC curve for IDA, ID, and ACDC in hypothyroid patients.

In group 1 (hypothyroid patients without iron deficiency taken as control), there were 28 males and 38 females with age range of 28–64 years. In group 2 (hypothyroid patients with IDA), there were 22 males and 44 females with age range of 18–61 years. In group 3 (hypothyroid patients with ID), there were 7 males and 5 females with age range of 43–60 years. In group 4 (hypothyroid patients with ACDC), there were 4 males and 6 females with age range of 43–63 years. After ROC analysis area under curve (AUC) of Ret-He for detecting IDA in hypothyroid patients was 0.96 at cutoff 28.5 pg with maximum specificity of 90% and sensitivity of 93%. AUC of Ret-He in hypothyroid patients with ACDC was 0.99 at cutoff 30.8 pg with sensitivity of 90% and specificity of 90%. AUC of Ret-He for diagnosing ID in hypothyroid patients was 0.97 at cutoff 31.7 pg with sensitivity of 91% and specificity of 70%. ROC curves for IDA, ID, and ACDC in hypothyroid patients have been compared as shown in Figure 1.

Hypothyroid patients in IDA, ID, and ACDC groups had significantly lower values of Ret-He than controls (*p* < 0.001). Mean values of Ret-He, Hb, ferritin, TSH, T3, and T4 in hypothyroid patients with IDA, ACDC, ID, and control are given in Table 1.

## 4. Discussion

The prevalence of thyroid disorders is quite high in Pakistan, but most hypothyroid patients with iron deficiency are misdiagnosed. Ret-He has been employed for the identification of iron deficiency in patients with different diseases, but, to our knowledge, this is one of the first studies which has focused on determining the effectiveness of Ret-He in diagnosing iron deficiency in hypothyroid patients. The study shows that Ret-He can be used as an effective single marker of iron deficiency in hypothyroid patients in different states. It is cost-effective, easily measured, performed on the same sample taken for complete blood count, and can identify iron deficiency in different states [19, 20]. We calculated Ret-He in 66 hypothyroid patients without iron deficiency taken as controls and 66 hypothyroid patients with IDA. Values of Ret-He were significantly lower in hypothyroid patients having IDA as compared to controls. Uçar et al. [21] conducted a study in which the value of Ret-He was significantly lower in iron deficient groups as compared to control. Our study also showed the value of Ret-He was significantly low in groups with iron deficiency in combination with anemia of chronic disorder and latent iron deficiency. A study was conducted by Wirawan et al. [22] on concordance between Ret-He and CHr in diagnosing iron deficiency in chronic kidney disease patients. They found Ret-He at cutoff 29.2 pg had a sensitivity and specificity of 95.5% and 94% in assessing the target of iron supplementation in hemodialyzed patients. A study performed by Gelaw et al. [23] shows significance of Ret-He in diagnosing iron deficiency in IDA and functional iron deficiencies. We found at cutoff 28.5 pg Ret-He had a sensitivity of 93% and specificity of 90% in diagnosing IDA. Research performed by Chinudomwong et al. [24] had comparable results. Their study demonstrated that Ret-He at cutoff 30 pg had 96% sensitivity and 97% specificity in diagnosing IDA. A study performed by Khan et al. [25] revealed Ret-He at cutoff <27.6 pg with sensitivity and specificity of 93.3% and 83.3% in diagnosing iron deficient states. Ret-He holds a special significance in anemia of chronic disease combined with iron deficiency as serum ferritin levels which are widely used in diagnosing iron deficiency that are altered due to chronic inflammation. We evaluated the role of Ret-He in identifying iron deficiency in combination with anemia of chronic disorder. Ret-He at cutoff 30.8 pg had sensitivity of 90% and specificity of 90% for identifying iron deficiency coexisting with anemia of chronic disorder. In cases where iron deficiency was present before developments of frank anemia, Ret-He at cutoff 31.7 pg had 91% sensitivity and 70% specificity. A study conducted by Singh et al. [26] evaluated the role of Ret-He in rheumatologic patients. They found Ret-He of <24 pg in IDA, 24–26.5 pg highly sensitive and specific in iron deficiency combined with anemia of chronic disorder, and >26.5 pg in anemia of the chronic disease group. Toki et al. [18] conducted a study on 211 patients aged 14–91 years of age. They classified patients into four groups: IDA group, ID group, control group, and anemia without ID group. The median Ret-He values were 22.3 (15.1–35.6 pg), 29.7 pg (19.2–34.9 pg), 34 pg (25.9–38 pg), and 32.5 pg (19.1–46.3 pg) in the IDA, ID, control, and anemia without ID. We had mean Ret-He of 21.35 pg in IDA. Dalimunthe and Lubis [27] calculated receiver operating characteristic (ROC) curve for Ret-He in iron deficient patients which revealed area under the curve of 0.818 (*p* < 0.0001), which is slightly lower than our study. Our study showed area under curve of 0.93. Their cutoff for diagnosing iron deficiency anemia was slightly higher than our results. They used 31.65 pg as cutoff Ret-He with 81.5% sensitivity and 61.6% specificity as compared to our cutoff Ret-He at 28.5 pg.

### 4.1. Limitations of Study

Despite being an important study, which assesses the importance of Ret-He in diagnosing iron deficiency in different states in hypothyroid patients, this study has some limitations. The role of Ret-He in diagnosing iron deficiency in hypothyroid patients with different hemoglobinopathies needs to be investigated as the number of patients having hemoglobinopathies specifically thalassemia is quite high in our country. Additionally, the number of hypothyroid patients in the study having ID and ACDC, the parameters suggesting the efficacy of Ret-He in diagnosing these states in hypothyroid patients, is quite small, and larger studies need to be conducted for further establishing the effectiveness of Ret-He in diagnosing ID and ACDC in hypothyroid patients. A further longitudinal limitation of this study is that it lacks follow up of patients after starting iron therapy.

## 5. Conclusion

In conclusion, Ret-He is an effective, rapid, inexpensive, and reliable parameter with very high sensitivity and specificity for ruling out concomitant iron deficiency in hypothyroid patients.

## Figures and Tables

**Table 1:** Biochemical parameters and thyroid profile in hypothyroid patients with IDA, ACDC, ID, and control.

Variables	Hypothyroid patients without iron deficiency and anemia (control) *n* = 66	Hypothyroid patients with iron deficiency and anemia (IDA) *n* = 66	Hypothyroid patients with iron deficiency without anemia (ID) *n* = 12	Hypothyroid patients with anemia of chronic disorder combined with iron deficiency (ACDC) *n* = 10
Ret-He (pg)	33.0 ± 2.1	21.0 ± 1.3	28.2 ± 0.2	25 ± 1.5
Hb (nmol/L)	8.98 ± 0.81	6.19 ± 0.1	9.10 ± 0.75	6.37 ± 0.2
Ferritin (ug/L)	144 ± 23.62	10.1 ± 2.0	123 ± 20.5	260.46 ± 55.75
TSH (mIU/L)	34.71 ± 0.15	36.0 ± 1.8	39 ± 2.1	40 ± 2.8
T3 (pmol/L)	2.97 ± 0.21	1.98 ± 0.13	2.01 ± 0.16	2.89 ± 0.22
T4 (pmol/L)	11.01 ± 0.32	9.41 ± 0.21	9.785 ± 0.23	10.2 ± 0.29

The data are expressed as mean ± S.D.

## Data Availability

The datasets generated and/or analyzed during the current study are not publicly available due to hospital policy on maintaining confidentiality and privacy of patient information but are available from the corresponding author upon request.

## Abbreviations

Ret-He: Reticulocyte hemoglobin equivalent
IDA: Iron deficiency anemia
ID: Latent iron deficiency
ACDC: Anemia of chronic disorder combined with iron deficiency.
